# Long-read amplicon denoising

**DOI:** 10.1093/nar/gkz657

**Published:** 2019-08-16

**Authors:** Venkatesh Kumar, Thomas Vollbrecht, Mark Chernyshev, Sanjay Mohan, Brian Hanst, Nicholas Bavafa, Antonia Lorenzo, Nikesh Kumar, Robert Ketteringham, Kemal Eren, Michael Golden, Michelli F Oliveira, Ben Murrell

**Affiliations:** 1 Department of Microbiology, Tumor and Cell Biology, Karolinska Institutet, Stockholm 17177, Sweden; 2 Department of Medicine, University of California, San Diego, La Jolla 92093, CA, USA; 3 Department of Biology, University of California, San Diego, La Jolla 92093, CA, USA; 4 Department of Pathology, Institute of Infectious Diseases and Molecular Medicine, Faculty of Health Science, University of Cape Town, Cape Town 7925, South Africa; 5 Department of Statistics, University of Oxford, Oxford OX1 3LB, UK

## Abstract

Long-read next-generation amplicon sequencing shows promise for studying complete genes or genomes from complex and diverse populations. Current long-read sequencing technologies have challenging error profiles, hindering data processing and incorporation into downstream analyses. Here we consider the problem of how to reconstruct, free of sequencing error, the true sequence variants and their associated frequencies from PacBio reads. Called ‘amplicon denoising’, this problem has been extensively studied for short-read sequencing technologies, but current solutions do not always successfully generalize to long reads with high indel error rates. We introduce two methods: one that runs nearly instantly and is very accurate for medium length reads and high template coverage, and another, slower method that is more robust when reads are very long or coverage is lower. On two Mock Virus Community datasets with ground truth, each sequenced on a different PacBio instrument, and on a number of simulated datasets, we compare our two approaches to each other and to existing algorithms. We outperform all tested methods in accuracy, with competitive run times even for our slower method, successfully discriminating templates that differ by a just single nucleotide. Julia implementations of Fast Amplicon Denoising (FAD) and Robust Amplicon Denoising (RAD), and a webserver interface, are freely available.

## INTRODUCTION

The Pacific Biosciences platform allows complex populations of long DNA molecules to be sequenced at reasonable depth. This has been used to study diverse viral populations (1–5), microbial communities (6,7), phage display libraries (8,9) and more.

PacBio SMRT sequencing generates extremely long reads (some >80 kb), with very high error rates (∼15%) (10). However, this length can be traded for accuracy. By ligating hairpin adapters that circularize linear DNA molecules, the sequencing polymerase can make multiple noisy passes around single molecules, and these can be collapsed into Circular Consensus Sequences (CCS) that have much higher accuracy (11).

When sequencing amplicons of a fixed length, the number of passes (i.e. the total raw read length divided by the amplicon length) is a primary determinant of the accuracy of a CCS read. The raw read length distribution has a long right tail, which means that the number of passes around each molecule, and consequently the CCS error rates, can vary substantially. Here, we confine our discussion to these CCS reads.

A critical feature of PacBio sequences is a high homopolymer indel rate. Laird Smith *et al.* (3) show that, for a 2.6 kb amplicon, under their quality filtering conditions, 80% of the errors are indels and 20% are substitution errors, and the indel errors are concentrated in homopolymer regions, increasing in rate with the length of the homopolymer. While high indel rates can be computationally challenging to deal with, since sequence alignment can be slow, they are favorable from a statistical perspective, because the errors appear in predictable places, making them more correctable (12).

Amplicon denoising (13–19) refers to a process that takes a large set of reads, corrupted by sequencing errors, and attempts to distill the noiseless variants and their frequencies. This has been extensively studied for short-read sequencing technology, but these approaches do not always generalize well to longer reads.

**Figure 1.**
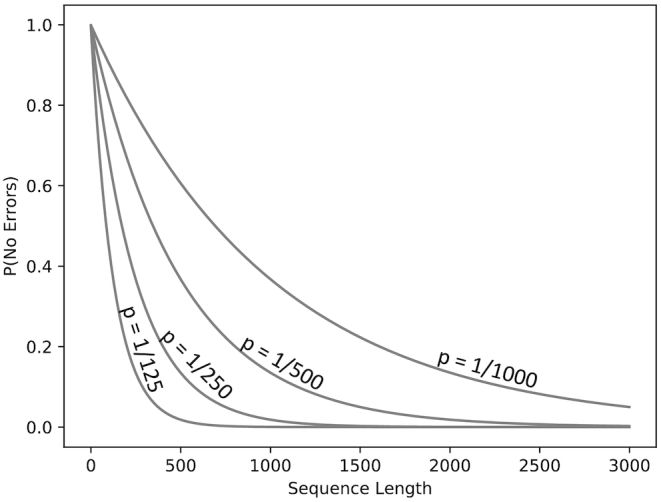
Under a simple error model, with constant per-base error probabilities (*P*), the probability that a sequence will have no errors decreases exponentially with the sequence length, with the slope of this decrease determined by *P*.

It is helpful to distinguish between two sequencing regimes: short and accurate (SA) and long and inaccurate (LI), and PacBio sequencing datasets can span both of these. For a given error rate, the probability of an observed read being noise free decreases exponentially with read length, and the error rate determines how precipitous this decline is (see Figure 1). For short, accurate reads, we can expect to have many noiseless representative reads in our dataset. Indeed, many Illumina amplicon denoising strategies (13,20) rely on this, and amount to simply identifying these reads using their relative abundance information. Shorter PacBio reads fall into this category as well. However, as the amplicon length increases, not only are there more opportunities for error, but the number of passes around each molecule decreases, increasing the per-base error rate. There may be variants that simply do not have any noiseless representatives, forcing us to abandon these ‘read-selection’ strategies of amplicon denoising in this long, inaccurate regime. We can only hope to reconstruct the noiseless reads by identifying a set of noisy reads that originate from the same variant, and averaging out their noise.

Previous approaches to this have used off-the-shelf clustering tools to render approximate reconstructions of the underlying population (3,21,22), but can be improved upon substantially.

Our strategy here embraces this SA/LI distinction, with one tool (FAD) that operates in the SA regime, and one (RAD) that operates in the LI regime. Both are implemented entirely in Julia, an emerging language for scientific computing.

## MATERIALS AND METHODS

### Overview

We present two methods: the fast amplicon denoiser (FAD) and the robust amplicon denoiser (RAD). FAD is designed for cases where an appreciable number of sequences are expected to be error free, and these can reliably serve as our inferred templates, avoiding any form of clustering or consensus calls, and exploiting abundance and neighborhood information to keep or reject templates. This method performs better for shorter amplicons, higher quality sequencing and better read-per-template coverage.

RAD is more complex, and designed for cases where very few reads are error free. This can occur in PacBio amplicon sequences when either amplicons are very long, with fewer passes per molecule, or for short movie lengths, reducing raw read lengths, or for older sequencing chemistries. RAD works in stages. We first employ a kmer-domain clustering approach, inspired by a non-parametric Bayesian procedure (23,24) to partition reads into clusters, followed by a recursive cluster refinement procedure (also in the kmer domain).

#### Kmer representation

For both RAD and FAD, we heavily exploit a kmer-based distance calculation. We first convert all sequences to their kmer counts. For all analyses here *k* = 6, representing each sequence as a vector of integers of length 4^*k*^. We then seek to approximate the pairwise edit distance between two sequences using these kmer frequency vectors.

While there exist sophisticated distance metrics based on kmer similarity (25), we opt for a simple approach that scales linearly with substitutions for low-divergences. Consider two identical sequences, with identical associated kmer vectors. When a random substitution is introduced, there will typically be ∼2*k* differences between the kmer vectors. So, our kmer approximation of edit distance is simply:

$$\begin{equation*} \mathcal {D}(A,B) = \frac{1}{2k}\sum _i^{4^k} (A_i-B_i)^2 \end{equation*}$$

**Figure 2.**
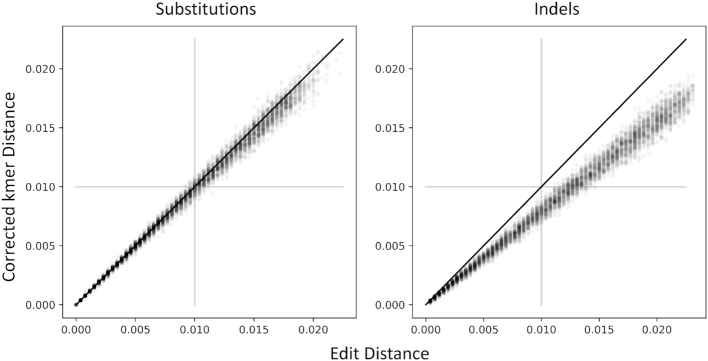
This distance approximates edit distance as mutations are introduced, starting from the 2599 bp NL4-3 HIV-1 env sequence. When only substitutions are introduced, edit distance is extremely well approximated. When indels are introduced, our kmer distance underestimates edit distance. This is desirable behavior when the sequencing error process is dominated by indels, because they will be downweighted in our distance function.

See Figure 2 for a demonstration of how this behaves, compared to edit distance. We can optionally scale this distance by dividing by the sequence length, to yield a per-base percentage difference.

### Fast amplicon denoising (FAD)

FAD is the simpler of the two algorithms, intended to work in low-noise scenarios. FAD proceeds by de-replicating reads, and sorting them by abundance, ignoring all reads that do not occur at least twice. FAD iterates through each read from largest to smallest, maintaining a set of accepted templates. When the current read is distant from all reads already included in the set by ≥1 bp (as calculated by our corrected kmer distance), then it is added to the set. If it is within 1 bp, then the abundances of the higher frequency template are considered when deciding to keep or discard the lower frequency template.

We first, however, correct the abundances by the expected proportion of error-free sequences. We convert the QV scores into error probabilities, and obtain an expected number of errors per sequence. We then evaluate the probability of each sequence having zero errors, and take the mean of this. For our 2.6 kb MVC dataset, this comes to 38%.

We take the most abundant template ≤1 bp from the current template, and we calculate the *P*-value for the size of a spurious offspring that differs at one base, under a Poisson error assumption, Bonferroni corrected for the average number of sites in the template. If this is ≤*α* (default: *α* = 0.01), then we reject the null hypothesis that we would obtain an offspring template this large by chance, and we include this template in the set.

Finally, we take all reads, and assign each read to an accepted template based on the minimum distance under the kmer approximation. These are used to compute the final frequencies for all reads.

### Robust amplicon denoising (RAD)

RAD is intended for high-noise scenarios, where we do not expect sufficient numbers of reads to be noise free for the strategy employed by FAD to succeed. We nevertheless aim to keep the computation time as low as possible, exploiting kmer distances extensively.

#### Dirichlet process means clustering

We wish to cluster our kmer frequency vectors. We need the algorithm to scale well with the input dataset size and the number of clusters, and we do not know how many clusters we have in advance (ruling out traditional options like *k*-means). But given that PacBio error rates per read are highly predictable from the quality scores, we can tell when two reads are sufficiently far that they cannot have originated from the same template. This distance threshold can be exploited by clustering approaches to automatically determine the number of clusters, and the ‘Dirichlet process means’ (DP-means) clustering approach (23) is ideal here.

It is frequently observed that k-means clustering can be derived as an expectation maximization algorithm for a finite mixture of isotropic Gaussians, where the variance of the Gaussians is sent to zero, forcing hard-assignments of elements to clusters (26). Similarly, DP-means can be derived as the limit of a sampling procedure for a non-parametric Bayesian Dirichlet process infinite Gaussian mixture model, where the variance is similarly driven to zero. This yields a surprisingly simple deterministic algorithm that uses a ‘radius’ parameter *λ* to control the number of clusters (23).

Briefly, the DP-means algorithm works by maintaining an array of centroids, and passing through the elements one at a time, computing the distance to all cluster centroids: if the distance between the element and any centroid is <*λ*, then assign the element to the cluster with the nearest centroid, and if not, seed a new cluster, using that element as the cluster centroid. After each pass through the elements, recompute the cluster centroids by averaging all the elements that are assigned to them. This iterates until convergence. See (23) for a technical description.

We use this algorithm to cluster our kmer vectors, using the scaled kmer distance, and a radius *λ* = 0.01, which is the error rate we typically use to retain .fastq reads in our data filtering steps. The ability to set the cluster radius to match the sequencing error rate is what makes DP-means an attractive clustering algorithm for this purpose.

The number of clusters is typically much lower than the number of reads. To reduce computation, after the first DP-means clustering pass yields a set of centroids, we cluster these centroids to construct a set of ‘meta-centroids’, and we compute, just once, the pairwise distance between all reads and all meta-centroids. Upon each subsequent iteration, we compute the pairwise distance between all current centroids and all meta-centroids, and we use the triangle inequality to avoid computing the read-to-centroid distances when we can deduce that they are >*λ*, reducing computation by a factor that depends on the template diversity.

#### Fine cluster splitting

Clustering reads using a radius equal to the error filtering cutoff can fail to distinguish variants that are very closely related. We therefore introduce a second layer of cluster refinement that directly seeks to split clusters that are different at any bases. Again, for computational efficiency, we remain in the kmer frequency vector domain to avoid sequence alignment.

Consider a cluster of a few closely related variants, each with multiple reads corrupted by sequencing noise (which has errors scattered at random bases). We attempt to suppress the noise by identifying the kmers that differ the most, and cluster just on these, with a very low clustering radius. To avoid splitting on homopolyer errors, we choose *M* (default *M* = 20) kmers with the largest variance, and search this set of high-variance kmers for kmer pairs that differ by a single homopolymer length edit, discarding these. We take the highest variance remaining *N* (default *N* = 6) kmers, and run DP-means clustering on this very low dimensional representation of the reads, with Euclidean distance, and a default radius of 1; i.e. if any reads differ at more than one of these kmers, we separate them. Please note that 1 bp difference should cause at least 6 kmers to differ, so this can split reads that differ by a single base.

This clustering step produces a ‘candidate’ cluster split, which we then decide to accept or reject, using the abundance information of these sub-clusters. If the original cluster gets fragmented into too many small clusters that fall below a size threshold, we reject the split. For this, we use the same Bonferroni corrected Poisson *P*-value approach as used in FAD. After splitting, we recurse, and continue splitting each sub-cluster until there is no evidence of heterogeneity.

#### Kmer-seeded alignment consensus

Unlike FAD, the clusters identified by RAD are not expected to have noise-free sequences associated with them. We thus rely on a consensus approach to infer these templates. We start by finding the sequence whose kmer vector is nearest to the cluster average kmer vector, which we take as a draft consensus. We then align all reads, pairwise, to this draft consensus. Using these pairwise alignment coordinates, we run a sliding window over the draft consensus, and when any blocks of this draft reference do not match the most common sub-sequence of the aligned reads, we replace that block of the draft reference with this modal value. We exploit kmer seeding (*k* = 30), and this approximate pairwise alignment algorithm scales linearly with sequence length.

If the amplicon spans a coding sequence, then Rifraf.jl (12) can be used to infer a frame-shift corrected template sequence, as long as a reference sequence with a trusted reading frame is available.

### A metric for comparing inferred to true templates

A population can be represented by a set of sequences, and their associated frequencies. We seek a metric that can be used to evaluate the reconstruction accuracy of an algorithm. A useful distance metric for evaluating reconstruction accuracy must be zero when the reconstructed sequences and frequencies are identical to the ground truth, should grow as the divergence between the two sets grows and should have a meaningful numerical interpretation. One attractive option here is the Earth Mover’s Distance, operating on the matrix of pairwise distances and frequencies. We have previously advocated this (21), but here we expand on this a little. We now refer to this as ‘Sequence Mutation Distance’ (*SMD*) and release SequenceMutationDistance.jl, which calculates this metric. A related approach, UniFrac (27,28), is commonly used to compare microbial communities, but UniFrac computes distances over a phylogeny, whereas SMD operates directly on the pairwise distance matrix, without phylogenetic assumptions.

Consider the ground truth sequences *A*, the inferred templates *B*, and a distance matrix *D* wherein *D*_*i, j*_ is the distance between *A*_*i*_ and *B*_*j*_ (here we use edit distance). Construct a flow matrix *F*, which is of the same shape as *D*, but *F*_*i, j*_ represents how much of *A*_*i*_ maps onto *B*_*j*_.

*SMD* can be defined as:

$$\begin{equation*} SMD = \min _F (\sum _i \sum _j F_{i,j} \times D_{i,j}) \end{equation*}$$

with respect to constraints:

$$\begin{equation*} \sum _{j} F_{i,j} = \text{freq}(A_i) \text{ and } \sum _{i} F_{i,j} = \text{freq}(B_j) \end{equation*}$$

where freq(*X*) is the frequency associated with variant *X*. This SMD score corresponds to the weighted average number of nucleotide changes per sequence required to convert *A* to *B*, finding the (possibly non-unique) minimum by optimizing over *F*. In our implementation, we use the Julia package JuMP.jl (29) to perform this optimization.

**Figure 3.**
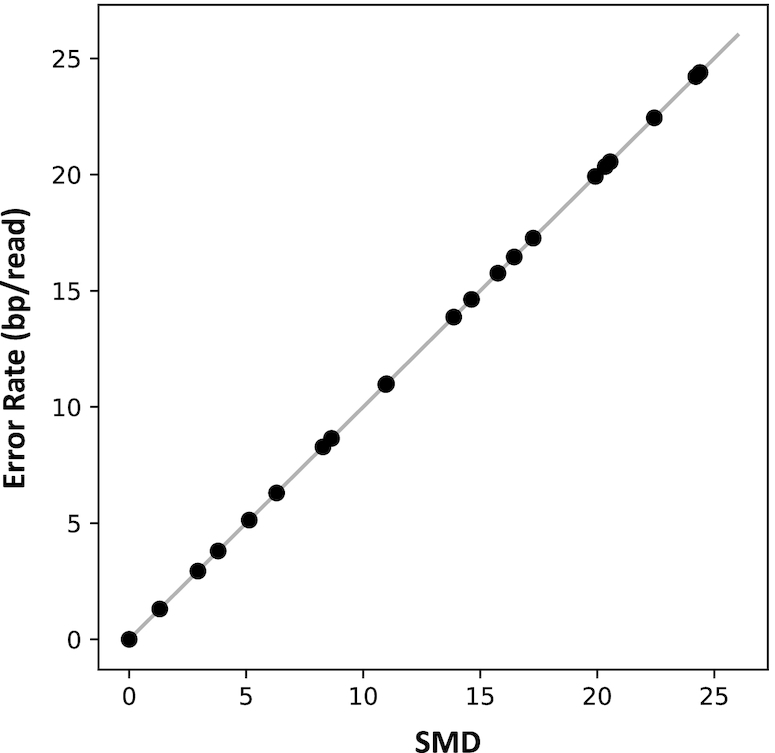
Sequence Mutation Distance (SMD) accurately approximates the average error rate, when computed between a set of templates, and a set of sequences that are derived from the templates by some noisy process.

This can be interpreted as the total error in the reconstruction per sequence. Indeed, if we compute the *SMD* between noisy sequences and the templates from which they were derived, we obtain a very precise estimate of the empirical error rate, biased only very slightly toward underestimation (see Figure 3).

The SMD score is optimized while respecting frequency constraints on both ground truth sequences *A*, and the inferred templates *B*. We can additionally derive two scores of interest: By relaxing the constraint on frequencies of *A*, we get *SMD*_*FP*_, which increases with the extent and frequency of false positives (i.e. reconstructed sequences that are absent from the ground truth). Similarly, by eliminating the constraint on *B*, we get *SMD*_*FN*_, a measure of false-negatives, which increases when our reconstructions are missing sequences that are present in the ground truth.

### Comparison methods

- VSEARCH: VSEARCH’s cluster-fast is run with an identity threshold of 0.99 (equivalent to our radius threshold of 0.01), and the consensus output is evaluated.
- USEARCH: USEARCH’s cluster-fast is run with identical parameters to VSEARCH, with an id threshold of 0.99. The ‘consensus output’ is used as inferred templates.
- Deep USEARCH: Similar parameters as the USEARCH method, but with the ‘max-accepts’ parameter set to 300 instead of the default, and the ‘max-rejects’ parameter set to 600 instead of the default, to cause USEARCH to search more aggressively for better matches during clustering.
- UNOISE: Fastx_uniques is run with a size output to dereplicate reads, followed by UNOISE3, using the ‘amplicon output’ (without chimera filtering) as inferred templates. Please note that the UNOISE documentation asserts that UNOISE is not designed for PacBio data.

All methods were run single-threaded on an AMD Ryzen 7 1700 processor @ 3.0 Ghz.

## RESULTS

We assembled five datasets to compare methods; two Mock Viral Communities (MVCs) each sequenced on a different PacBio instrument, and three datasets simulated using the PacBio sequence simulator developed in (21):

- 2.6 kb MVC: We used a number of closely related HIV envelope clones available in our laboratory, each comprising an *env* sequence embedded in a pLenti-III-HA plasmid (Addgene). To construct a ground truth clustering for these reads, we attempted to amplify 96 single clones in single wells, using paired forward and reverse primers that uniquely identify the well, sequenced on the RS-II using P6/C4 chemistry, with 6-h movie lengths. CCS reads were inferred with PacBio’s CCS algorithm (v3.0), and filtered at the 1% accuracy threshold (3). From this, we recovered clonal sequences from 80 wells. The consensus of reads from each well is taken as the ground truth sequence. When inferring templates using RAD, FAD and other methods, we first trim off the barcodes from the .fastq reads to ensure the true clustering is obscured. The full dataset had ∼18k reads, but we also subsampled datasets of 10k, 5k and 2k to investigate lower template coverage (which could occur when multiplexing samples).
- 5 kb MVC: This used the same strategy and population as the 2.6 kb MVC, but we amplified a longer (4.8 kb) stretch of the pLenti+*env* construct. This was sequenced on a PacBio Sequel, using the 3.0 chemistry, with 10-h movie lengths. All preprocessing steps were identical to the 2.6 kb dataset. We recovered clonal sequences from 83 wells. The full dataset had ∼86.6k reads, but we also subsampled datasets of 40k, 20k, 10k and 5k to investigate lower template coverage (which could occur when multiplexing samples). Since this dataset was so large, to avoid interminable processing we imposed a 5-h compute time limit for all methods.
- EnvSim low diversity: This is a simulated dataset. We obtained templates and frequencies from a run of the Full Length Envelope Analysis pipeline (FLEA) (21) on donor P018 (3). The ‘low diversity’ dataset (17782 reads) is simulated from the P018 ‘V06’ time point, ∼6 months post-infection, representing a challenging dataset of low diversity. We include an unfiltered dataset, as well as one filtered at a 1% expected error threshold.
- EnvSim high diversity: As for the previous simulation, but using the 33 months post-infection templates and frequencies, which had higher diversity, simulating 11798 reads. The error profiles for the P018 datasets were generated to match P5/C3 chemistry (the previous generation), and have a higher mean error rate than our P6/C4 and 3.0 datasets, which impacts the relative performance of the methods. We again include an unfiltered dataset, as well as one filtered at a 1% expected error threshold.
- 9 kbSim: We simulated low-diversity evolution of 9 kb templates, starting from the full-length nl43 plasmid, with random mutations (including indels), generating 32 closely related templates. Frequencies were simulated from a uniform distribution, resulting in 5916 reads. We matched the error rates in the simulated reads to those from a 9 kb plasmid sequence (data not shown), and these are substantially lower than the ∼2.6 kb amplicons, primarily due to the amplicon length. Here we include unfiltered, 1% filtered and 2% filtered datasets.

Template sequence phylogenies and summary statistics for these datasets are depicted in Supplementary Figure SI1.

### Performance

**Figure 4.**
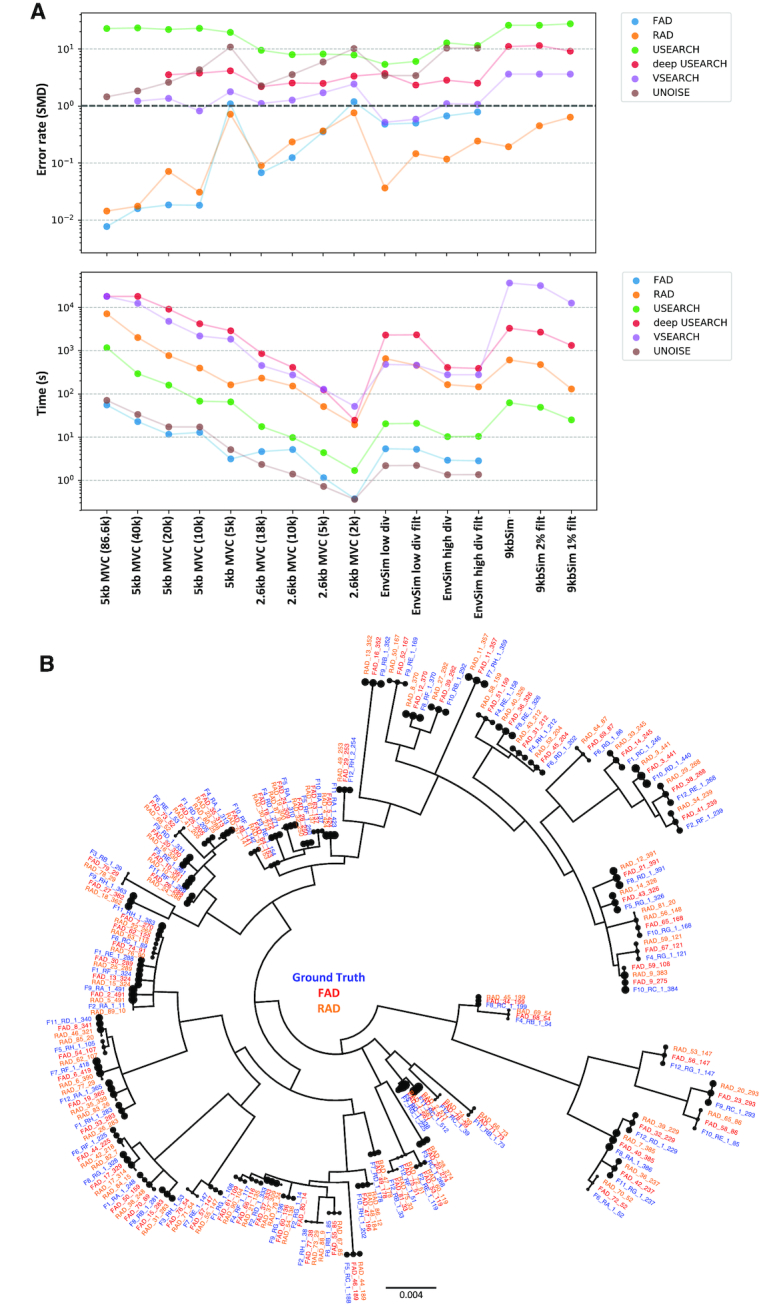
(**A**) Error rates (measured by Sequence Mutation Distance) of reconstructions against ground truth for a number of datasets. 2.6 kb MVC is a real sequencing dataset, using primer barcodes to obtain the ground truth clustering. P018 (∼2.6 kb) comprises reads simulated *in silico* from a set of templates obtained from an HIV+ donor, from a low diversity, early time point, and a later, more diverse, time point. The 9 kb dataset comprises a set of closely related templates, with long reads simulated from these, using a higher error rate profile. The dashed horizontal gray line shows the threshold for an expected error rate (by SMD) of 1 bp per sequence. Also shown below are run times of the various methods. (**B**) From the 2.6 kb MVC full dataset, we show a phylogeny depicting the ground truth templates, as well as the inferred templates for FAD and RAD.

See Figure 4 for accuracy (SMD scores) and timing results. These SMD scores are not normalized by sequence length and can be interpreted as the per-sequence error rate. So, an SMD of 1.0 means that there is, on average, 1 bp incorrect in each sequence. False positive and false negative SMDs are shown in Supplementary Figure SI2, and all numerical results are shown in Supplementary Tables SI1 and SI2.

USEARCH (with default parameters) and UNOISE are fast, but inaccurate. In many cases, USEARCH has an accuracy similarly to the SMD of uncorrected reads. UNOISE, as expected, is not well suited to long-read datasets with higher error rates. Deep USEARCH, modified for a more extensive search during clustering, is slower and more accurate than USEARCH. The timing difference can be dramatic: from a minute for USEARCH, to nearly an hour for deep USEARCH, when inferring templates from the unfiltered 9 kb dataset. VSEARCH has intermediate accuracy, with SMDs as low as 0.51 for the P018 low-diversity dataset. VSEARCH runs in times comparable to deep USEARCH for 2.6 kb datasets, but becomes very slow for 9 kb datasets, taking 10 h on the slowest dataset without a timeout condition.

FAD (like UNOISE) does not complete on the 9 kb datasets, because it requires an appreciable proportion of error-free reads. FAD is extremely fast on all 2.6 kb datasets, never taking longer than 6 s, and completed 5 kb MVC (the largest dataset) in under a minute. FAD is the most accurate method on the full 2.6 kb MVC dataset, with an SMD of 0.068 (which translates to a per-base error rate of 1 in ∼38 800). As expected, FAD’s performance degrades as the template coverage decreases, and as the error rates increase (the simulated 2.6 kb datasets had higher error rates than 2.6 kb MVC). Strikingly, FAD was also the most accurate method on the 5 kb dataset, across all subsampling sizes besides the 5000 read version, where RAD became more accurate. On the full dataset, FAD obtained an SMD error rate of 0.0077 errors per read, which is entirely driven by fractional differences in the variant frequencies compared to the ground truth. This shows that FAD can take dramatic advantage of the accuracy improvements facilitated by the Sequel 3.0 chemistry.

RAD is always faster than deep USEARCH, and faster than VSEARCH on all-but-two datasets, and has especially well controlled run times for the 9 kb datasets (where it takes 10 min compared to e.g. VSEARCH’s 10 h). RAD, however, stands out as being consistently accurate across all datasets, with results close to FAD in the low-noise 2.6 kb MVC and 5 kb MVC datasets, but with clearly superior results across the noisier regimes. The closest competitor not proposed here, VSEARCH, has substantially higher SMD scores than RAD on all datasets, with accuracies ranging from 2.4× to 69.7× worse (on the 5 kb MVC 5000 and 5 kb MVC 50 000 datasets, respectively), and is substantially slower.

While many template sequences in 2.6 kb MVC were closely related to each other, there were two template sequences that differed by just a single base. Despite this, RAD and FAD were able to reconstruct both variants.

The 5 kb MVC dataset demonstrates that the Sequel 3.0 chemistry is a dramatic improvement over the RS-II P6/C4. Despite reads being nearly twice as long as the 2.6 kb MVC dataset, the per-read accuracy was sufficient for an approach such as FAD to be competitive, suggesting many reads with zero errors. 4.8 kb templates are more accurately reconstructed with RAD and FAD from 10 000 Sequel reads than 2.6 kb templates are from 18 000 RS-II reads. This is despite these SMD scores representing per-sequence error rates, and so longer reads should be more error prone given the same per-base error rate. Interestingly, the other methods appear less able to take advantage of the improvements in the sequencing chemistry. The constant region in this dataset also afforded an examination of the homopolymer error rates, both in the original CCS reads, as well as post-denoising (see Supplementary Figure SI3).

Please note that none of these results should be taken as a criticism of USEARCH or VSEARCH’s clustering, as these algorithms were not designed with this problem in mind, nor of UNOISE (which explicitly states that it is not for PacBio reads).

### Single-chain Fv

**Figure 5.**
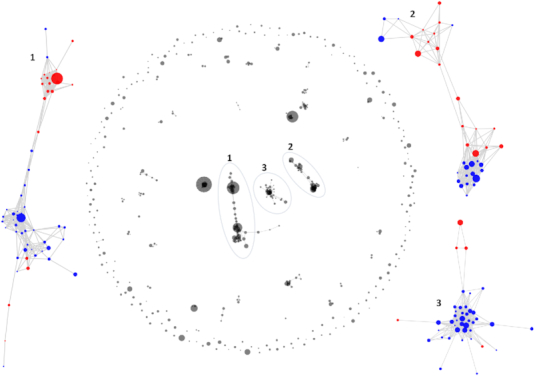
Visualizing the structure of the FAD-denoised variants from the single-chain Fragment variable phage library, after 4 rounds of selection. Variant frequency is depicted with bubble size, and variants with ≤2.36% corrected kmer distance (the minimum distance between any pre- and post-selection variant) are connected in the network. We also show the largest 3 connected components, coloring each variant depending whether the scFv linker was short (blue) or long (red).

To investigate amplicon denoising behavior under more extreme diversity than is possible with mock community datasets, we applied our approach to a single-chain Fragment variable (scFv) phage display library (30). Given the short read lengths ( 830 bp), we used FAD, which recovered 512 variants. While this dataset has no ground truth, after four rounds of phage display enrichment we might expect that it is enriched for intact reading frames, so we can compare the proportion of stop codons in the original CCS sequences (40.6%) versus the denoised variants (3.7%), suggesting a dramatic denoising effect. We can also use our fast corrected kmer distance to compare the denoised variants in the post-selection library with those in the pre-selection library, showing that not a single variant post-selection is within 2% divergence from any read in the pre-selection library (minimum: 2.36%). This is consistent with extreme heterogeneity in the pre-selection library, which is expected. Since these variants aren’t related by descent, we cannot use phylogenetics to visualize them, but we can use NextGenSeqUtils.jl and other tools from the Julia environment to visualize the community network structure of the post-selection variants, using the minimum pre-to-post distance to construct a meaningful connectivity threshold (see Figure 5, and see https://nextjournal.com/Murrell-Lab/scfv-fad-analysis/ for a NextJournal notebook showcasing this analysis). Here, a ‘community’ of variants highlights where distinct variants with similarities at the sequence level have been jointly enriched during the selection process, providing evidence that such variants are enriched due to similar functional properties, rather than just stochastically increasing in frequency through successive population bottlenecks. Note that immune repertoires from unsorted cells typically exhibit much greater diversity than this dataset, and will require substantial depth to achieve sufficient per-template coverage for these approaches to be successful, but reconstructing long templates from lower diversity cell populations (e.g. after antigen sorting) should be possible.

## DISCUSSION

We have presented two algorithms, FAD and RAD, for denoising long PacBio amplicons. While we intend to use these tools primarily for applications in virology, which has motivated our choice in datasets, there is no reason why they cannot be used in any long-read amplicon sequencing domain, especially for metagenomics (e.g. 16S or even the entire 16S-23S rRNA region). RAD and FAD can both be run without any specialist computing hardware—a standard laptop suffices even for large Sequel datasets.

In addition to the methods considered here, we also attempted to use the Long Amplicon Analysis tool (https://github.com/PacificBiosciences/pblaa), but this did not appear to be suitable for datasets of the complexity of our MVCs (see Supplementary Figure S5).

The appreciable proportion of error-free reads in the 5 kb MVC dataset was surprising, and can likely be attributed to improvements in both sequencing chemistry and PacBio’s circular consensus algorithms. In fact, we expected FAD, which was inspired by short-read amplicon denoising approaches, to fail completely. The fact that such strategies can be used for PacBio reads was independently demonstrated by Callahan *et al.* (31) for a 1.5 kb amplicon, further supporting the prospect of resolving amplicon sequencing datasets at single nucleotide resolution.

The distribution of read accuracies can vary due to factors that one has no control over (such as the distribution of homopolymer lengths in the system being studied), factors that one can stipulate (such as the PacBio movie length), but also a number of factors that one can only partially influence, such as the proportion of intact molecules or how close one is to the optimal loading concentration. For these reasons, only speculative guidelines can currently be provided about what amplicon length may successfully be approached with these techniques. Our experience suggests that FAD should be effective up to at least 5 kb, and additional Sequel datasets (not analyzed here) suggest that the average number of errors per read for a 9 kb Sequel amplicon is roughly one-third of those used in the 9 kb simulation here (which was modeled on RS-II accuracies). So, while amplicons of 9 kb will not be amenable to FAD analyses, we speculate that RAD will be able to denoise amplicons longer than 9 kb. Additionally, FAD reports a statistic that should be predictive of the number of noiseless reads, and recommends when RAD should be used instead.

Our 5 kb MVC analysis suggests that amplicon sequencing on a Sequel can be productively multiplexed, and here we saw that 10 000 reads provided reconstructions comparable to the 86k reads of the original dataset. This could vary depending on the template length, and the number of templates, and requires further exploration.

With this paper we release four Julia packages: NextGenSeqUtils.jl, RobustAmpliconDenoising.jl, DPMeansClustering.jl and SequenceMutationDistance.jl (hosted at https://github.com/MurrellGroup), which should be a helpful contribution to the Julia next-generation sequencing ecosystem. We also provide a web server (link maintained at: https://github.com/MurrellGroup/webservers) for convenient analyses. A .fastq CCS file is uploaded, and filtering options can be selected. Either FAD or RAD is run, and the inferred templates, as well as a number of visualizations (see Supplementary Figure SI4), are provided. Finally, we also provide multiple use examples, including additional features such as demultiplixing with custom primer barcodes, in computable NextJournal notebooks (https://nextjournal.com/Murrell-Lab) that allow analyses to be ‘remixed’ and run in the cloud.

The algorithms could potentially be improved along multiple dimensions:

- Automatically determining the optimal method for amplicon denoising: we currently use a simple heuristic to choose which of RAD or FAD should be used. This uses the QV scores to obtain an estimate of the expected number of error free reads, and it uses the proportion of identical reads. If both of these are sufficiently high, use FAD, but if either is low, we recommend RAD. The details of what counts as high or low require further exploration on additional datasets.
- Test other kinds of sequencing data: future work should compare amplicon denoising methods on datasets from a wider range of sources, spanning a range of length and template diversity.
- Using error rates when clustering: since the error rates are highly predictable, the distance between a read and a centroid could be adjusted by the expected error in each, which could result in more accurate clustering.
- Using error rates when splitting: per-base error rates could also be exploited during cluster splitting for both FAD and RAD, potentially improving accuracy.
- A hybrid algorithm that combines the characteristics of FAD and RAD, first reconstructing as deeply as possible using FAD, and then polishing with RAD. We intend to explore this in the near future.
- Parallelization: We could gain run-time improvements by parallelizing some components of our model. The simplest of these would be the RAD consensus step, where each consensus can be executed on a different thread.

Additional extensions may be domain specific. For example, chimera filtering (32–34) is not useful in domains like HIV, where extensive biological recombination produces the same signals as artificial chimeras. However, this could be useful in other domains, and should be implemented. Finally, we note that our approach does not tolerate read fragments (from e.g. shotgun sequencing), and we do not expect that it is extensible to such cases.

## CONCLUSION

The advent of accurate long-read denoising approaches shifts the developmental burden away from data processing. Going forward, the primary impediment to extending the length of amplicons that can be sequenced is the design of PCR strategies that can successfully amplify very long templates.

## Supplementary Material

gkz657_Supplemental_File
