# Supplementary material for: Long-read amplicon denoising
Source: Nucleic Acids Res. 2019 Aug 16;47(18):e104. doi: 10.1093/nar/gkz657 (PMC6765106; doi:10.1093/nar/gkz657)
Supplement: gkz657_Supplemental_File [file gkz657_supplemental_file.pdf]

Dataset	Method	Time	SMD	SMD_FP	SMD_FN
5kb MVC 86599	USEARCH	1169.0	22.812	7.3653	6.1677
5kb MVC 86599	deep USEARCH	Timeout	NaN	NaN	NaN
5kb MVC 86599	VSEARCH	Timeout	NaN	NaN	NaN
5kb MVC 86599	FAD	55.498	0.0077137	0.0	0.0
5kb MVC 86599	RAD	7133.4	0.014399	0.0029335	0.0
5kb MVC 86599	UNOISE	70.939	1.4496	0.00079428	0.0
5kb MVC 40000	USEARCH	293.35	23.372	3.7582	7.1793
5kb MVC 40000	deep USEARCH	Timeout	NaN	NaN	NaN
5kb MVC 40000	VSEARCH	12458.0	1.2193	0.095898	0.12648
5kb MVC 40000	FAD	22.867	0.015875	0.0	0.0
5kb MVC 40000	RAD	2011.3	0.0175	0.001625	0.0
5kb MVC 40000	UNOISE	33.465	1.8378	0.0	0.0
5kb MVC 20000	USEARCH	159.84	21.951	3.9358	9.9698
5kb MVC 20000	deep USEARCH	9121.8	3.5393	0.4861	0.9172
5kb MVC 20000	VSEARCH	4777.8	1.3597	0.12944	0.25425
5kb MVC 20000	FAD	11.608	0.01845	0.0	0.0
5kb MVC 20000	RAD	769.56	0.071354	0.0012001	0.05045
5kb MVC 20000	UNOISE	17.248	2.6022	0.0	0.091
5kb MVC 10000	USEARCH	68.006	23.008	8.1575	10.393
5kb MVC 10000	deep USEARCH	4191.2	3.7632	1.0779	1.2936
5kb MVC 10000	VSEARCH	2189.1	0.81906	0.021755	0.0974
5kb MVC 10000	FAD	12.865	0.0182	0.0	0.008
5kb MVC 10000	RAD	395.6	0.0308	0.0	0.0212
5kb MVC 10000	UNOISE	17.15	4.318	0.0	1.0498
5kb MVC 5000	USEARCH	65.392	19.518	3.9581	8.9802
5kb MVC 5000	deep USEARCH	2887.7	4.1407	0.90558	1.47
5kb MVC 5000	VSEARCH	1846.0	1.7781	0.24476	0.5332
5kb MVC 5000	FAD	3.1385	1.0898	0.0	0.7732
5kb MVC 5000	RAD	162.38	0.7172	0.0	0.5356
5kb MVC 5000	UNOISE	5.1391	10.808	0.0	7.7732
2.6kb MVC 18112	FAD	4.6446	0.067856	0.017613	0.003644
2.6kb MVC 18112	RAD	231.54	0.090437	0.02606	0.017778
2.6kb MVC 18112	UNOISE	2.3348	2.2587	0.0	0.11468
2.6kb MVC 18112	USEARCH	17.547	9.482	0.50841	1.4506
2.6kb MVC 18112	deep USEARCH	857.42	2.184	0.54416	1.1057
2.6kb MVC 18112	VSEARCH	453.12	1.1084	0.080107	0.23907
2.6kb MVC 10000	FAD	5.1706	0.1243	0.0064	0.0566
2.6kb MVC 10000	RAD	152.03	0.2346	0.0214	0.1496
2.6kb MVC 10000	UNOISE	1.397	3.5554	0.0	0.6545
2.6kb MVC 10000	USEARCH	9.7907	7.9491	0.80864	3.2107
2.6kb MVC 10000	deep USEARCH	410.93	2.5291	0.7151	1.3111
2.6kb MVC 10000	VSEARCH	274.41	1.2691	0.16172	0.443
2.6kb MVC 5000	FAD	1.1525	0.353	0.0	0.2822
2.6kb MVC 5000	RAD	50.947	0.36581	0.0066026	0.1552
2.6kb MVC 5000	UNOISE	0.72427	5.9239	0.0	1.5628
2.6kb MVC 5000	USEARCH	4.3795	8.1488	0.86011	2.7488
2.6kb MVC 5000	deep USEARCH	124.19	2.4908	0.76001	1.489
2.6kb MVC 5000	VSEARCH	128.17	1.7074	0.18667	0.822
2.6kb MVC 2000	FAD	0.37589	1.19	0.0	0.7805
2.6kb MVC 2000	RAD	19.465	0.76009	0.015523	0.442
2.6kb MVC 2000	UNOISE	0.36397	10.153	0.0	6.325
2.6kb MVC 2000	USEARCH	1.6917	7.8397	1.0257	4.6495
2.6kb MVC 2000	deep USEARCH	24.617	3.3399	0.84859	2.076
2.6kb MVC 2000	VSEARCH	51.529	2.4249	0.35006	1.0615

Table S1. Single-threaded timing results and SMD scores for all real datasets.

Dataset	Method	Time	SMD	SMD_FP	SMD_FN
EnvSim low div	FAD	5.3841	0.48032	0.0	0.42931
EnvSim low div	RAD	655.05	0.03656	0.0	0.025869
EnvSim low div	UNOISE	2.1893	3.4121	0.0	1.5971
EnvSim low div	USEARCH	20.399	5.366	0.050423	3.9028
EnvSim low div	deep USEARCH	2298.2	3.7077	0.82039	2.0704
EnvSim low div	VSEARCH	482.14	0.51757	0.035696	0.15465
EnvSim low div_filt	FAD	5.2401	0.50128	0.0	0.42931
EnvSim low div_filt	RAD	456.84	0.14595	0.0	0.11028
EnvSim low div_filt	UNOISE	2.215	3.4121	0.0	1.5971
EnvSim low div_filt	USEARCH	20.868	6.0444	0.96581	4.394
EnvSim low div_filt	deep USEARCH	2328.0	2.3296	0.24087	0.90552
EnvSim low div_filt	VSEARCH	462.75	0.58692	0.047954	0.12867
EnvSim high div	FAD	2.9212	0.67011	0.0	0.63307
EnvSim high div	RAD	163.95	0.11725	0.00076291	0.10731
EnvSim high div	UNOISE	1.3507	10.344	0.0	6.2534
EnvSim high div	USEARCH	10.276	12.777	0.13379	1.7105
EnvSim high div	deep USEARCH	408.66	2.8349	0.32861	1.0642
EnvSim high div	VSEARCH	277.84	1.0991	0.020323	0.23945
EnvSim high div_filt	FAD	2.8312	0.78546	0.0	0.63307
EnvSim high div_filt	RAD	145.37	0.24308	0.0	0.10637
EnvSim high div_filt	UNOISE	1.3594	10.344	0.0	6.2534
EnvSim high div_filt	USEARCH	10.409	11.482	0.062843	1.4801
EnvSim high div_filt	deep USEARCH	388.91	2.5098	0.24889	1.046
EnvSim high div_filt	VSEARCH	277.86	1.0702	0.011554	0.23674
9kb	FAD	2.6043	NaN	NaN	NaN
9kb	RAD	609.8	0.1932	0.0	0.18932
9kb	UNOISE	2.3922	NaN	0.0	NaN
9kb	USEARCH	62.531	26.005	9.8841	23.835
9kb	deep USEARCH	3294.7	11.045	5.8296	5.595
9kb	VSEARCH	36730.0	3.6239	0.21184	0.6408
9kb_filt2	FAD	2.5854	NaN	NaN	NaN
9kb_filt2	RAD	476.86	0.45012	0.0	0.18932
9kb_filt2	UNOISE	2.1348	NaN	0.0	NaN
9kb_filt2	USEARCH	49.098	25.99	7.3187	23.762
9kb_filt2	deep USEARCH	2682.7	11.426	5.9051	5.9508
9kb_filt2	VSEARCH	31920.0	3.6239	0.21184	0.6408
9kb_filt1	FAD	1.0061	NaN	NaN	NaN
9kb_filt1	RAD	129.85	0.63611	0.0	0.054429
9kb_filt1	UNOISE	1.0254	NaN	0.0	NaN
9kb_filt1	USEARCH	25.106	27.614	10.333	25.468
9kb_filt1	deep USEARCH	1322.3	9.1448	5.2663	5.8352
9kb_filt1	VSEARCH	12610.0	3.6223	0.21184	0.6408

Table S2. Single-threaded timing results and SMD scores for all simulated datasets.

2.6kb MVC

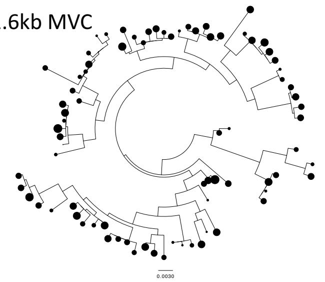

Source:	**RS-II (P6/C4)**
Mean (Length):	2629.6
Pairwise Diversity (%):	2.15
Average Nearest (%):	0.44
# templates:	80
Mean (frequency):	226.4
STD (frequency):	124.8
Mean errors/read:	5.85

5kb MVC

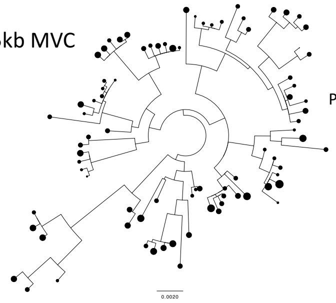

Source:	**Sequel (3.0)**
Mean (Length):	4857.7
Pairwise Diversity (%):	1.15
Average Nearest (%):	0.24
# templates:	83
Mean (frequency):	1043.4
STD (frequency):	413.8
Mean errors/read:	10.9

EnvSim low div

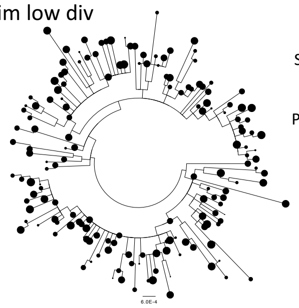

Sim Template Source:	**P018 early**
Mean (Length):	2609.05
Pairwise Diversity (%):	0.447
Average Nearest (%):	0.089
# templates:	165
Mean (frequency):	107.8
STD (frequency):	54.08
Mean errors/read:	9.06

EnvSim high div

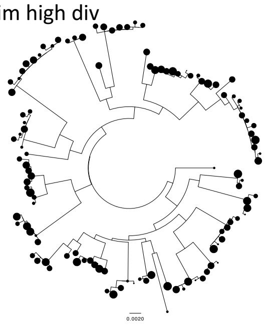

Sim Template Source:	**P018 late**
Mean (Length):	2573.7
Pairwise Diversity (%):	2.209
Average Nearest (%):	0.131
# templates:	115
Mean (frequency):	102.59
STD (frequency):	51.44
Mean errors/read:	8.86

9kbSim

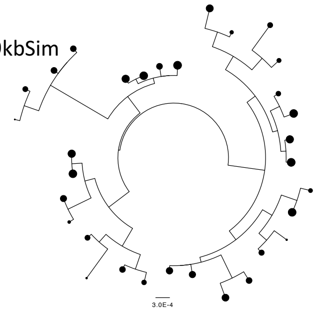

Sim Template Source:	**Mutated NL43**
Mean (Length):	9698.75
Pairwise Diversity (%):	0.361
Average Nearest (%):	0.066
# templates:	32
Mean (frequency):	184.9
STD (frequency):	81.51
Mean errors/read:	111.57

Fig. S1. Five test datasets. The first two are Mock Viral Communities, sequenced on the older RS-II and the newer Sequel, respectively. See main text for more detailed descriptions. Here we show maximum likelihood phylogenies of the template sequences, along with summary statistics: the mean read length, the mean pairwise diversity, the average distance from each template to its closest neighbour, the number of templates, the average template frequency, the standard deviation over template frequencies, and the mean number of errors per read.

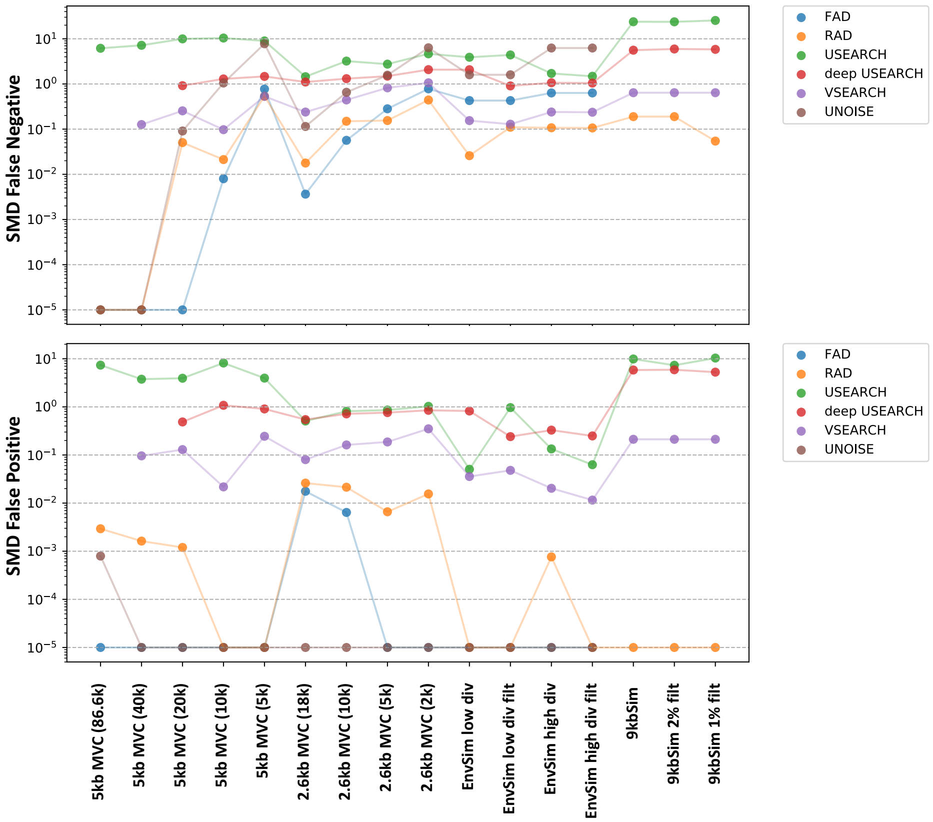

Fig. S2. False Negative (top) and False Positive (bottom) SMD scores of reconstructions against ground truth for a number of datasets. Values of 0 are set to 10^{-5} for the log transform.

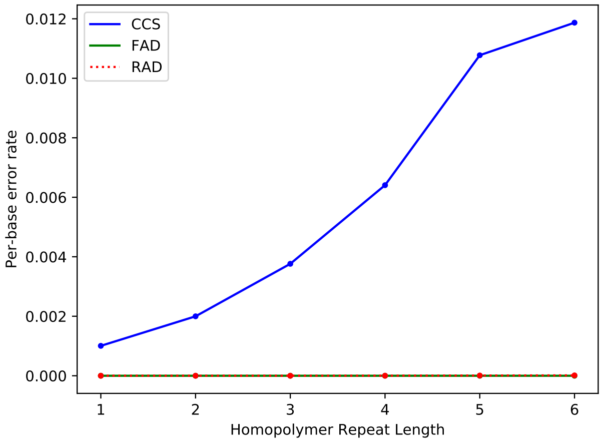

Fig. S3. Homopolymer error profile, constructed from CCS reads, and RAD, and FAD variants, against a 2.3kb region of the 5kb MVC dataset that is constant across all variants, allowing unambiguous accuracy quantification. Errors are "per-base". CCS error (ie. without denoising) rates range from 1 in 1000 for non-homopolymer repeats up to 1 in 100 for 6bp homopolymers. RAD and FAD per-base error rates are averaged as though each read was replaced with the denoised version of itself to obtain equivalent per-base error rates (ie. they are averages, weighted by variant frequency).

A

B

C

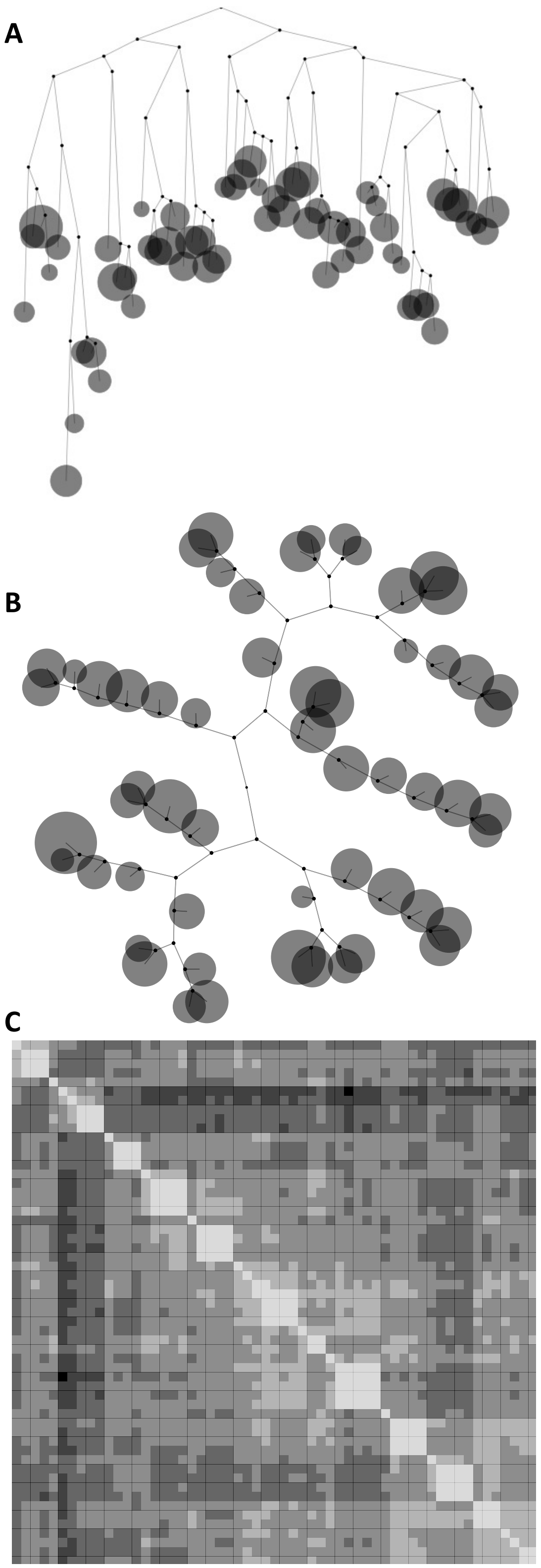

Fig. S4. Interactive visualization of inferred templates and their frequencies is available in multiple layouts on the RAD/FAD webserver. Neighbour joining phylogenies are inferred from fast corrected kmer distance matrices, and displayed in traditional phylogeny format (A), as well as D3 force directed graph layout (B). Sequence names are shown interactively. We also display distance matrices (C). Together, these allow a rapid assessment of the diversity and population structure of the inferred template sequences.

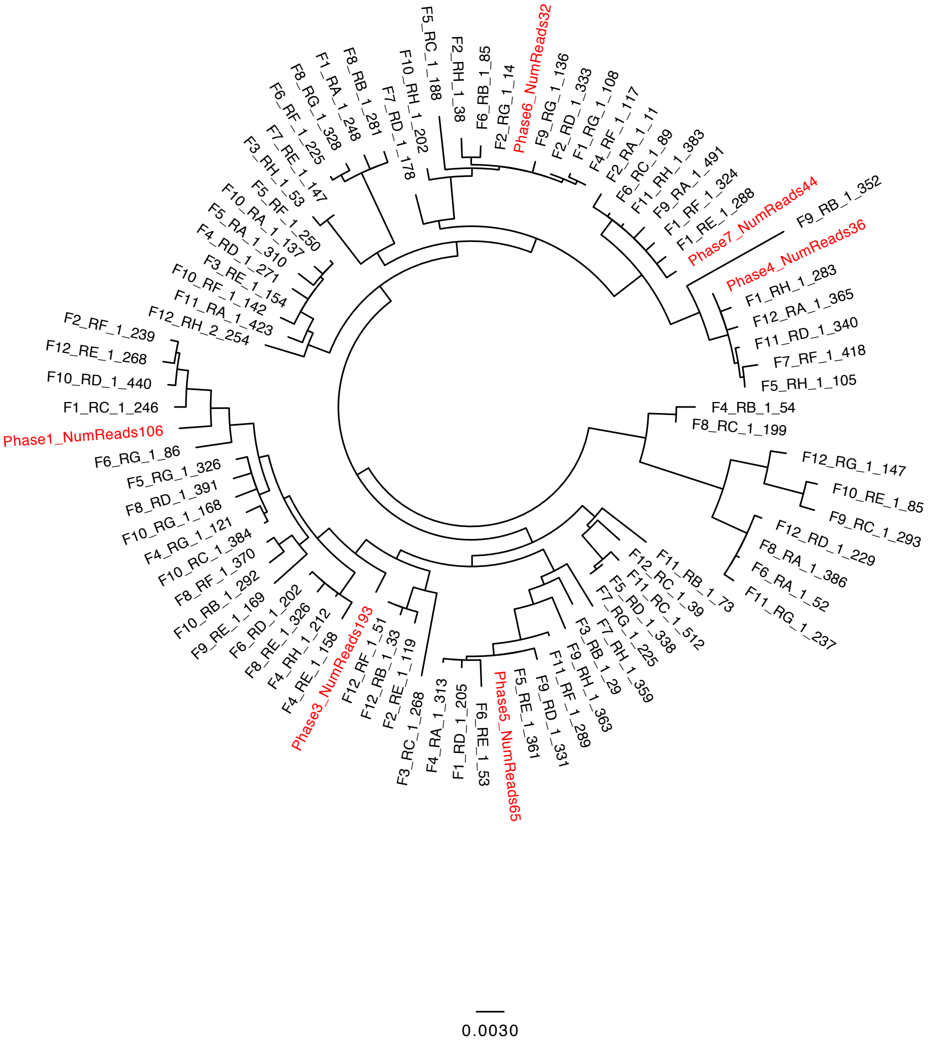

Fig. S5. LAA recovers very few variants in a mock virus community dataset.The Long Amplicon Analysis (LAA) tool operates on raw data, which means we cannot digitally remove the variant barcodes from the .fastq CCS sequences, which prevents a full and fair comparison. But even allowing LAA the advantage of processing these sequences with the barcodes present, it does not appear to be suited for datasets with extensive diversity, and recovers only 6 variants (red) when run on default settings (besides length, which was adjusted to encompass the amplicon length). The situation was not improved by modifying the LAA run parameters. Note that the extent of LAA's applicability is not yet clear, and this analysis is not intended as a general criticism of LAA.
